# Molecular analysis of *Pseudomonas aeruginosa* strains isolated from cystic fibrosis patients

**DOI:** 10.1038/s41598-021-95034-2

**Published:** 2021-07-29

**Authors:** Dariusz Jarych, Ewa Augustynowicz-Kopec, Agnieszka Iwanska, Pawel Parniewski, Marta Majchrzak

**Affiliations:** 1 Institute of Medical Biology PAS, Lodz, Poland; 2 Department of Microbiology, National Tuberculosis and Lung Diseases Research Institute, Warsaw, Poland

**Keywords:** Microbiology, Molecular biology, Medical research

## Abstract

*Pseudomonas aeruginosa* is a severe bacterial pathogen. Due to the genetic flexibility among strains, chronic airways infection can lead to mortality among cystic fibrosis (CF) patients. It is essential to develop patient-specific therapy which will rely on phenotypic and genomic diversity. The primary objective of this study was to assess the genomic variability of *P. aeruginosa* strains, using two different molecular techniques for tracking the epidemiological transmissions. This study applied a multiple-locus variable-number tandem-repeat (VNTR) analysis (MLVA) for an efficient genotyping of clinical *P. aeruginosa* strains isolated from CF patients and compared results with a TRS-PCR typing. The percentage similarity analysis was performed using the categorical multi-state coefficient and UPGMA method. Based on the MLVA and TRS-PCR group assessment, 43 *P. aeruginosa* strains/variants were detected among the 63 clinical isolates from eight CF patients. The study of *P. aeruginosa* isolates has revealed that during chronic bacterial infections, CF patients harbor different *P. aeruginosa* strains or variants within the same host over the years. *P. aeruginosa* genotypes diversity may result from infection with several strains and result from a microevolution process of an initially acquired strain. The TRS-PCR method proposed in this work can complement the MLVA scheme. It can also be used as a preliminary method for genetic typing of P. aeruginosa isolates in CF patients.

## Introduction

Cystic fibrosis (CF) is a rare, incurable, chronic, and fatal autosomal recessive genetic disease^1^. According to the World Human Organization, in the European Union, 1 in 2000–3000 newborns is affected by CF. The cause of the disease is mutations in the *cftr* gene encoding an adenosine 3’,5’-cyclic monophosphate (cAMP)-regulated Cl^−^ channel, among which deletion of phenylalanine at position 508 (F508del) defects intracellular processing and trafficking^2^ and occurs for ~ 66–70% of the Caucasian population^1,3^ and 53–57% of Polish CF patients^3^. Lung failure, blocked with thick, sticky secretions, is the cause of premature death at a very young age. According to the CF Foundation's National Patient Registry, the median age of survival for a person with CF is currently 33.4 years.

Defective mucociliary clearance and function of the cystic fibrosis transmembrane conductance regulator (CFTR) in airway epithelium and submucosal glands plays a crucial role in the lung colonization by the opportunistic Gram-negative bacteria *Pseudomonas aeruginosa* causing pneumonia*.* Increased morbidity and mortality of CF patients are connected with chronic airways inflammation with this pathogen, which persists in the CF lung because of its genetic flexibility and ability to adapt to changing environments^4^.

After *P. aeruginosa* acquisition from environment or from person-to-person transmission, motile planctonic form changes its features and establishes a chronic infection by forming mucoid colonies, altered phenotypes and increasing antibiotic resistance^5,6^. During the chronic infection, *P. aeruginosa* strains form a biofilm composed of exopolysaccharides (alginate, Pel, and Psl), polypeptides, and extracellular DNA, increasing tolerance to antibiotics and helps bacteria to resist phagocytosis and to be insensitive to the innate and adaptive human immune system^7^.

The antimicrobial multidrug resistance (MDR) depends on the overexpression of efflux pumps, the loss of the outer membrane porin (OprD), and increased expression of β-lactamases-coding genes: ESBLs (extended-spectrum β-lactamases) and MBLs (metaloβ-lactamases) associated with mobile genetic elements (MGE) which promote resistance spread during the process of horizontal gene transfer and to grow the acquisition of multidrug resistance among *P. aeruginosa* isolates^8^.

It is crucial to diagnose and set an appropriate patient treatment early. Some treatment regimens include oral ciprofloxacin in combination with nebulized colistin or employing inhaled tobramycin. Since chronic infection cannot be eradicated, treatment is effective at an early stage of infection. Improper treatment or late diagnosis leads to increased mortality^5,9^. Some reports suggest that after curing the primary infection, the chronic infection was caused by another *P. aeruginosa* strain^10^. There are also reports suggesting that CF patients have been infected with several bacterial strains ^11^.

To perform *P. aeruginosa* strain identification at an early stage of infection, several genotyping methods have been applied for the strains’ discrimination. The most commonly used methods are amplified fragment length polymorphism (AFLP)^12^, random amplified polymorphic DNA (RAPD)^6,12^, ribotyping^13^, multilocus sequence typing (MLST)^6^, multiple-locus variable-number tandem-repeat (VNTR) analysis (MLVA)^6,13–15^, pulsed-field gel electrophoresis (PFGE)^6,12,14,16^. Repetitive-element-based PCR, such as enterobacterial repetitive intergenic consensus (ERIC)-PCR and BOX-PCR^16^ target bacterial non-coding repetitive sequences and are highly conserved across species^17^. They are simple and fast, but they lack interlaboratory reproducibility^12^. A TRS-PCR is another method used for the efficient genotyping of clinical pathogenic strains^18^. This method relies on the presence of microsatellites trinucleotiderepeat sequences^19^, such for example GTG, CAC, CGG, CCG, or CTG in bacterial genomes. TRS-PCR profiles have the potential to serve as valuable markers for phylogenetic and epidemiological studies since the different trinucleotide repeat sequence elements vary in their copy number and distribution in bacterial genomes^18–22^. Comparing to other methods, a TRS-PCR has higher reproducibility and discriminatory index than ERIC-PCR^19^ and MLVA^21^. A PFGE offers a highly discriminatory method for genetic fingerprinting, but it is time-consuming and difficult to standardize and interpret^14^. Due to the high cost of MLST analysis, its usage in small diagnostic laboratories is unaffordable^14^. Given the low cost and ability of MLVA, this technique allows interlaboratory comparisons making this method widely used for genotyping *P. aeruginosa*^13^.

In this study, we applied a widely used multiple-locus variable-number tandem-repeat (VNTR) analysis (MLVA) for an efficient genotyping of clinical *P. aeruginosa* strains isolated from CF patients and, for the first time, we extended the obtained results with data from TRS-PCR profiling.

## Results

### Multiple loci VNTR analysis of the clinical P. aeruginosa isolates

Nine repetitive elements loci scheme (six minisatellites and three microsatellites) was selected to perform the MLVA. The method successfully distinguished 63 *P. aeruginosa* isolates used in this study. In general, the allelic profile for each strain can be described by a code corresponding to the number of repeats at the selected VNTR based on the PCR product size in the following order: ms061, ms172, ms211, ms213, ms214,ms217, ms222, ms207, and ms209, as it is shown for example for the DK01 isolate: 11–6-1–1-2–5-4–3-4. The repeatability analysis was performed by conducting the PCR of all VNTRS loci for 20 isolates in duplicate (data not shown). The analysis showed compliance of received profiles among duplicates. MLVA distinguished 26 unique groups among the isolates, including **14 singletons** and **12 complexes** differing at not more than one VNTR locus**.**

### TRS-based PCR genotyping

In order to compare the discriminatory power of VNTR genotyping, isolates were also typed by TRS-PCR. A total of 63 clinical *P. aeruginosa* isolates were applied for genotyping, and all isolates within the collection were typeable by the CGG- and GTC-PCR. Isolates were grouped according to genotype after a clustering analysis was performed, as described above. The DNA of each strain generated a complex fingerprint pattern used for building separate (CGG)_4_ and (GTC)_4_-based PCR dendrograms. Considering the reproducibility value, up to 27 CGG-based PCR clusters and 10 GTC-based PCR clusters could be assigned to isolates. Based on this analysis, each unique profile was assigned a class number^24^. Based on the TRS-class assessment, 40 unique TRS-PCR groups can be obtained, which differ in at least one CGG- or GTC-class, resulting in 25 singletons and 15 complexes (Table 1), which showed more distinct types than MLVA based genotyping.

**Table 1:** The comparison of MLVA and TRS-PCR genotyping results for 63 *P. aeruginosa* isolates from 8 CF patients.

Isolate	Date of Isolation	ms061	ms172	ms207	ms209	ms211	ms213	ms214	ms217	ms222	MLVA group	MLVA strain/variant*	CGG-class	GTC-class	TRS-group	Total strain/variant
DK01	09/2013	11	6	1	1	2	5	4	3	4	16	DK-01	16	3	21	DK-SV01
DK02	01/2014	10	5	17	3	3	1	1	4	2	7	DK-02	9	9	13	DK-SV02
DK03	01/2014	10	5	17	3	3	1	1	4	2	7	DK-02	9	9	13	DK-SV02
DK04	08/2014	10	5	11	3	3	1	1	4	2	5	DK-02*	7	9	11	DK-SV03
DK05	01/2018	10	5	11	3	3	1	1	4	2	5	DK-02*	8	10	12	DK-SV04
DK06	10/2018	10	5	11	3	3	1	1	4	2	5	DK-02*	9	9	13	DK-SV02
DK07	10/2018	10	5	14	3	3	1	1	4	2	6	DK-02*	9	9	13	DK-SV02
DP01	01/2013	10	6	2	1	5	13	24	3	3	9	DP-01	22	5	30	DP-SV01
DP02	01/2014	10	6	2	1	5	13	24	3	3	9	DP-01	20	5	27	DP-SV02
DP03	01/2014	10	6	2	1	5	13	24	3	3	9	DP-01	20	7	28	DP-SV03
DP04	01/2015	10	6	2	1	5	13	24	3	3	9	DP-01	21	7	29	DP-VS04
DP07	04/2018	12	7	1	3	6	4	4	3	3	23	DP-02	27	1	40	DP-SV05
DPA01	04/2012	11	8	12	2	4	3	4	1	1	21	DPA-01	3	3	3	DPA-SV01
DPA02	10/2012	11	7	8	2	5	9	16	3	3	18	DPA-02	23	5	33	DPA-SV02
DPA03	02/2013	11	7	8	2	5	9	16	3	3	18	DPA-02	23	5	33	DPA-SV02
DPA04	01/2014	11	7	8	2	5	9	16	3	3	18	DPA-02	23	7	34	DPA-SV03
DPA05	10/2017	11	8	2	2	3	5	4	3	4	19	DPA-03	17	1	22	DPA-SV04
DPA07	04/2018	11	9	8	2	3	6	3	5	1	22	DPA-04	23	1	32	DPA-SV05
DPA08	04/2018	11	7	8	2	5	9	16	3	3	18	DPA-02	23	7	34	DPA-SV03
DPA09	01/2019	11	8	2	2	7	1	1	4	3	20	DPA-05	17	1	22	DPA-SV06
JK01	06/2014	10	6	1	3	1	5	5	2	4	8	JK-01	11	3	16	JK-SV01
JK02	10/2015	10	6	1	3	1	5	5	2	4	8	JK-01	12	3	17	JK-SV02
JK03	06/2018	10	6	1	3	1	5	5	2	4	8	JK-01	10	3	14	JK-SV03
JK04	10/2018	10	6	1	3	1	5	5	2	4	8	JK-01	5	3	7	JK-SV04
JK05	01/2019	10	6	1	3	1	5	5	2	4	8	JK-01	5	3	7	JK-SV04
KP01	02/2013	11	6	5	1	5	9	20	3	3	17	KP-01	22	5	30	KP-SV01
KP02	09/2013	12	7	5	2	24	3	4	2	1	24	KP-02	1	3	1	KP-SV02
KP03	02/2014	12	7	5	2	24	3	4	2	1	24	KP-02	1	3	1	KP-SV02
KP05	02/2016	12	7	5	2	24	3	4	2	1	24	KP-02	2	3	2	KP-SV03
KP06	01/2017	10	6	3	1	2	4	4	3	4	11	KP-03	18	3	23	KP-SV04
KP07	11/2017	10	6	3	1	2	4	4	3	0	10	KP-03*	19	3	25	KP-SV05
KP08	01/2018	12	7	5	2	24	3	4	2	1	24	KP-02	4	3	5	KP-SV06
KP09	09/2018	10	6	3	1	2	4	4	3	4	11	KP-03	18	4	24	KP-SV07
KP10	10/2018	10	6	3	1	2	4	4	3	4	11	KP-03	19	4	26	KP-SV08
LA02	02/2013	10	8	3	1	6	3	4	2	1	14	LA-01	6	4	10	LA-SV01
LA03	04/2014	10	8	3	1	6	3	4	2	1	14	LA-01	6	1	8	LA-SV02
LA04	02/2017	10	8	3	1	6	3	4	2	1	14	LA-01	6	1	8	LA-SV02
LA05	02/2017	10	8	3	1	24	3	4	2	1	15	LA-01*	6	1	8	LA-SV02
LA06	04/2018	10	3	3	1	2	3	4	2	3	4	LA-02	5	1	6	LA-SV03
LA07	04/2018	10	3	3	1	2	3	4	2	3	4	LA-02	5	1	6	LA-SV03
LA08	07/2018	10	3	3	1	2	3	4	2	3	4	LA-02	4	1	4	LA-SV04
LA09	07/2018	15	8	3	1	24	3	4	2	1	26	LA-01*	6	1	8	LA-SV02
LA10	08/2018	0	5	3	1	2	4	1	4	3	1	LA-04	6	2	9	LA-SV05
PA01	11/2013	12	7	11	2	24	4	4	2	1	25	PA-01	10	4	15	PA-SV01
PA02	11/2014	10	7	14	1	4	4	4	1	1	13	PA-02	15	4	20	PA-SV02
PA05	02/2016	10	6	11	2	5	13	24	3	3	12	PA-03	25	6	37	PA-SV03
PA06	08/2016	10	7	14	1	4	4	4	1	1	13	PA-02	16	3	21	PA-SV04
PA07	08/2016	10	7	14	1	4	4	4	1	1	13	PA-02	14	3	19	PA-SV05
PA08	02/2017	10	6	11	2	5	13	24	3	3	12	PA-03	25	6	37	PA-SV03
PA10	03/2018	10	7	14	1	4	4	4	1	1	13	PA-02	14	3	19	PA-SV05
PA11	03/2018	0	6	11	2	5	13	24	3	3	2	PA-04	22	6	31	PA-SV06
PA12	11/2018	10	7	14	1	4	4	4	1	1	13	PA-02	13	3	18	PA-SV07
PA13	11/2018	10	7	14	1	4	4	4	1	1	13	PA-02	16	3	21	PA-SV04
WE01	05/2014	9	7	3	1	1	3	3	3	4	3	WE-01	25	3	36	WE-SV01
WE02	02/2015	9	7	3	1	1	3	3	3	4	3	WE-01	25	3	36	WE-SV01
WE03	03/2017	9	7	3	1	1	3	3	3	4	3	WE-01	25	3	36	WE-SV01
WE04	05/2017	9	7	3	1	1	3	3	3	4	3	WE-01	26	3	38	WE-SV02
WE05	05/2017	9	7	3	1	1	3	3	3	4	3	WE-01	24	3	35	WE-SV03
WE06	04/2018	9	7	3	1	1	3	3	3	4	3	WE-01	26	3	38	WE-SV02
WE07	04/2018	9	7	3	1	1	3	3	3	4	3	WE-01	26	8	39	WE-SV04
WE08	06/2018	9	7	3	1	1	3	3	3	4	3	WE-01	26	8	39	WE-SV04
WE10	02/2019	9	7	3	1	1	3	3	3	4	3	WE-01	26	8	39	WE-SV04
WE11	02/2019	9	7	3	1	1	3	3	3	4	3	WE-01	26	3	38	WE-SV02

Isolate—single colony obtained from the selective agar plates of plated sputum samples from the patient in the following periods; ms061, ms172, ms207, ms209, ms211, ms213, ms214, ms217, ms222—mini- and microsatellites loci; MLVA group -unique groups based on the similar VNTR profile; MLVA Strain/Variant—unique *P. aeruginosa* isolate analyzed based on the MLVA; CGG-class, GTC-class—profiles assigned based on the fingerprint similarity comparisons; TRS-group—unique groups based on the similar CGG- and GTC-class; Total Strain/Variant—unique *P. aeruginosa* isolate analyzed based on the MLVA- and TRS-group assessment.

### MLVA and TRS-PCR combined similarity analysis

Based on the MLVA and TRS-PCR group assessment, 43 *P. aeruginosa* strains/variants were detected among the 63 clinical isolates from eight CF patients. Strains with the same TRS-PCR and MLVA groups were considered identical. The analysis showed that isolates with the same genotype originated from the same patients. The comparison of the MLVA and TRS-PCR genotypes among CF patients is present in Table 1.

For a DK patient, two strains (DK-01, DK-02) were identified (groups 16, 7) and two variants of DK-02 strain (DK-02*) using the MLVA method (groups: 5,6) and four different types of isolates using the TRS-PCR method (groups: 21, 13, 11, 12). MLVA and TRS-PCR methods combined allowed us to find four isolates among seven samples collected from this patient (DK-SV01-DK-SV04).

For a DP patient, two strains were identified (DP-01, DP-02) with the MLVA method (groups 9, 23) and five isolates using the TRS-PCR method (groups 30, 27, 28, 29, 40). MLVA and TRS-PCR methods combined allowed us to find five isolates among five samples collected from this patient (DP-SV01-DP-SV05.

For a DPA patient, five strains were identified (DPA-01-DPA-05) with the MLVA method (groups 21, 18, 19, 22, 20) and six isolates using the TRS-PCR method (groups 3, 33, 34, 22, 32, 34). MLVA and TRS-PCR methods combined allowed us to find six isolates among eight samples collected from this patient (DP-SV01-DP-SV06).

For a JK patient, one strain was identified (JK-01) with the MLVA method (group 8) and four isolates using the TRS-PCR method (groups 16, 17, 14, 7). Therefore, MLVA and TRS-PCR methods combined allowed us to find four isolates among five samples collected from this patient.

For a KP patient, three strains (KP-01-KP-03) were identified (groups 17, 24, 11) and one variant (KP-03*) using the MLVA method (group 10). MLVA and TRS-PCR methods combined allowed us to find eight isolates among nine samples collected from this patient (KP-SV01-KP-SV09).

For a LA patient, three strains (LA-01-LA-03) were identified (groups 14, 4, 1) and two variants of LA-01 strain (groups 15, 26). MLVA and TRS-PCR methods combined allowed us to find five isolates among nine samples collected from this patient (LA-SV01-LA-SV05).

For a PA patient, four strains (PA-01-PA-04) were identified using the MLVA method (groups 25, 13, 12, 2). MLVA and TRS-PCR methods combined allowed us to find seven isolates among ten samples collected from this patient (PA-SV01-PA-SV07).

For a WE patient, only one strain (WE-01) was identified using the MLVA method (group 3). MLVA and TRS-PCR methods combined allowed us to find four isolates among ten samples collected from this patient WE-SV01-WE-SV04).

The concordance between MLVA and TRS-PCR typing methods was analyzed by the Wallace coefficient. Wallace _TRS-PCR→MLVA_ = 0.576 and Wallace _MLVA→TRS-PCR_ = 0.186, meaning that if two strains are in the same cluster by TRS-PCR, they have about 58% chance of having the same MLVA type, while conversely, this is about 19% chance. This emphasizes the fact that TRS-PCR is more discriminatory than MLVA typing.

## Discussion

Chronic airway infections of CF patients have offered a unique view into a microevolutionary adaptation of *P. aeruginosa*. *P. aeruginosa* undergoes a high level of genetic and phenotypic heterogeneity during prolonged and persistent infections, resulting from clonal pathoadaptive variants with different features compared to the initially acquired strain^25^. The specific adaptations of *P. aeruginosa,* such as overproduction of alginate and loss of motility, contribute to developing various subpopulations within a single patient. *P. aeruginosa* clonal strains more effectively avoid the immune reactions, are more resistant to antimicrobial drugs, and are less metabolically active than the primary strains^7,26^. Initially acquired strain may diversify by de novo mutations and the composition of the accessory genome and can persist in the host organism for many years. This progressive microevolution results in less virulent strains through losses of LPS, type II and III secretion system, or even genomic islands^27^. The knowledge of the genotypic differences of these chronic adapted isolates is advantageous to provide information and tools for a better approach to treating infections in CF patients.

This study provides the genotypic analysis of *P. aeruginosa* isolates collected from eight adult CF patients at the National Tuberculosis and Lung Diseases Research Institute in Warsaw between 2012 and 2019. The primary objective of this study was to assess the genomic variability of *P. aeruginosa* strains, using a combination of the two different molecular techniques to increase the methodology sensitivity.

The data we have obtained indicate the need for a patient-specific approach that therapy will consider on the phenotypic and genomic diversity of *P. aeruginosa* in the lung of CF patients. Our results confirm the idea that conditions in the CF lung support strain diversification and genetic variation. The existence of strain diversity during chronic lung infections caused by *P. aeruginosa* may serve as a marker of disease progression and be used in novel therapies.

Our study has not shown a shared strain among patients, suggesting that the risk of patient-to-patient transmission is low. During the 8-year study period, we observed that the *P. aeruginosa* CF patients harbored unique strains that confirm that these patients most likely acquired strains from environmental sources. Although patient-to-patient transmission of strains has been reported in some clinics, we have not found evidence of cross-infection. Researchers have been conducting interesting experiments for the last few years and have shown the occurrence of aggressive and transmissible strains of *P. aeruginosa* in patients attending CF centers worldwide^28^. As a result, some transmissible clones may be more virulent than the usual infecting types, possibly resulting in poor prognoses for the patients^26,29^. Our study suggests that the hygiene procedures at the National Tuberculosis and Lung Diseases Research Institute are successful and prevent cross-transmission between CF patients. However, as this study was carried out using a small number of patients and strains, this statement cannot be generalized for all CF populations, and further studies must be done to confirm it.

This study confirms that MLVA and TRS-PCR are very robust genotyping techniques that can be applied to the systematic survey of P. aeruginosa isolates in CF patients and demonstrate that both methods effectively characterize clinical isolates of *P. aeruginosa.* Although the TRS-PCR method has previously been applied for molecular genotyping of other pathogens^21–23^, this technique was used for the first time to demonstrate the colonization and differentiation of *P. aeruginosa* in adult Polish patients with cystic fibrosis.

Our results suggest that DNA typing tools such as MLVA combined with TRS-PCR may play an important role in routine epidemiological surveillance and the identification of the source of transmission of *P. aeruginosa* in CF patients. This study suggests that both TRS-PCR and MLVA are suitable, inexpensive, fast, reproducible, and discriminatory DNA typing tools for effective epidemiological surveillance of potential transmissible *P. aeruginosa* isolates between patients with CF. TRS-PCR provided results similar to those obtained by the MLVA typing. However, it seems that this method is, in some cases, more discriminatory compared to MLVA, and Wallace coefficient values indicate this. Such genotyping methods combined with additional genetic, phenotypic, or other epidemiological data can play an essential role in making significant decisions on infection control issues. Rapid diagnostic determination of isolate genotype and phenotype is essential to avoid the spread of dangerous super-resistant *P. aeruginosa* strains; however, epidemiological data should always be considered when deciding whether genetically related strains are also epidemiologically related.

Examination of *P. aeruginosa* strains also revealed that a single bacterial colony cannot represent the entire MLVA/TRS-PCR type, as different genotypes of *P. aeruginosa* can simultaneously colonize patients. The analysis of few colonies from multiple sampling from the same period may be more representative of an entire genotype. The obtained results showed the differentiation of some strains with identical MLVA profiles (strains isolated from JK and WE patients) by applying the TRS-PCR technique. Instead, it may indicate that these are clones that have changed due to microevolution and that these are not distinct strains. We also found strains isolated from the same patient that differed in both MLVA and TRS-PCR profiles. It may indicate that the patient has become infected with many strains simultaneously or has acquired new ones during the primary infection. The coexistence of genetically distinct subpopulations in the lung of a single patient complicates treatment. The different *P. aeruginosa* strains/clones detected among a single patient might contribute to the lack of correlation between microbiology test results and the clinical status of a patient. Moreover, one or a few isolates will not accurately represent the behavior of an entire, chronically infecting population of these bacteria. During the first three years of colonization, two or even more *P. aeruginosa* clones can be observed^27^.

The present study showed a relatively high polymorphism among *P. aeruginosa* isolates from the same hospital ward. In conformity with other studies, the results obtained suggest that *P. aeruginosa* diversify within the same host and variate in the genotype over the years^30,31^. We believe that the genotypic identification and characterization of *P. aeruginosa* strains isolated from CF patients could be an accurate way to study infection epidemiology and allows for a more accurate assessment of prophylactic, therapeutic, and control measures against this infection.

In our opinion, the TRS-PCR method proposed in this paper can either complement the MLVA scheme or be used as a preliminary, fast, cheap, and repeatable method for determining the diversity of *P. aeruginosa* isolates.

## Methods

### Characteristics of patients and clinical isolates

Sixty-three *P. aeruginosa* isolates were obtained between 2012 and 2019 from a total of eight patients from the Institute of Tuberculosis and Lung Diseases in Warsaw, Poland, and used in this study. All these clinical strains originated from adult patients (≥ 18 years old) with CF and were sampled in the hospital during subsequent infections. Table 2 shows the characteristics of patients included in this study.

**Table 2:** Characteristics of the eight patients diagnosed at the institute of tuberculosis and lung diseases between 2012 and 2019.

Mean age, years (SD)	30,3 (10,9)
Median age of diagnosis, years (SD)	8,8 (7,8)
Sex, males/females	3/5
**CFTR classification**^**a**^	
del F508 homozygous, N (%)	3 (37,5)
del F508 heterozygous, N (%)	3 (37,5)
Other, N (%)	2 (25,0)
**Comorbidities**	
Pancreatic insufficiency (%)^b^	6 (75,0)
Cystic fibrosis-related diabetes (%)^c^	4 (50,0)
FEV_1_% predicted; mean (SD)	45,3 (18,9)
FVC_1_% predicted; mean (SD)	61,8 (14,4)
BMI; median (IQR)	18,9 (18,3–19,8)

^a^Homozygous: both alleles containing the delta F508 mutation; Heterozygous: one allele containing the delta F508 mutation; Other: alleles containing mutations that are different.

^b^Pancreatic insufficiency: use of pancreatic enzymes.

^c^Cystic fibrosis-related diabetes: use of insulin.

### Bacterial growth and genomic DNA isolation

Sputum samples were collected from each patient and plated on the selective media for the isolation of *P. aeruginosa* including: Columbia Blood Agar(bioMérieux, Marcy l'Etoile, France) and MacConkey Agar (bioMérieux). Identification of *P. aeruginosa* was carried out by routine microbiological methods (oxidase, pigment production, growth at 42 °C), and to confirm identification, we were using the commercial identification system, VITEK®2 (bioMérieux) and MALDI-TOF (Matrix Assisted Laser Desorption Ionization Time-of-Flight, Bruker Daltonics). Single colonies were picked up from the agar plate and cultured overnight in a 5 ml Luria–Bertani (LB) liquid medium at 37 °C with agitation (120 rpm).On the next day, bacterial cell cultures were transferred to a new 5 ml LB liquid medium using an inoculation loop and cultured at 32 °C or 37 °C for 18–48 h with agitation at 120 rpm, until OD 0.9–1.0 was reached. 1 ml of bacterial cell culture was centrifuged (16,000 RCF, 10 min) and the supernatant was decanted. Genomic DNA was isolated from the bacterial pellet using a GenElute Bacterial Genomic DNA Kit (Sigma-Aldrich, St. Louis, MO), according to the manufacturers’ protocol, at an average concentration of 50 ng/µl. The quantity and purity of each genomic DNA sample were measured by spectrophotometry at 260 nm (BioPhotometer, Eppendorf, Germany). The DNA samples were diluted to 10 ng/µl and then used.

### MLVA genotyping

Multi-locus variable-number tandem repeat analysis (MLVA) was performed for all 63 *P. aeruginosa* isolates used in this study, according to the nine variable-number tandem-repeat (VNTR) loci scheme proposed previously by Turton J.F., *et al*^23^*.* Primer sequences for amplification of ms61, ms172, ms207, ms209, ms211, ms213, ms214, ms217, and ms222 loci were selected from Onteniente L. et al*.*^15^ and Vu-Thien H., et al*.*^13^. The PCR was optimized and performed in a total volume of 25 µl consisted of 20 ng of DNA, 1 × *Taq* polymerase reaction buffer (Invitrogen by Life Technologies, CA, USA), 1 U *Taq* polymerase (Invitrogen by Life Technologies, Waltham, USA), 1.5 mM of MgCl_2_ (for ms211 1.0 mM and for ms222 2.0 mM of MgCl_2_ were used, respectively), 200 µM of each deoxynucleotide, 6% dimethyl sulfoxide (DMSO) and 0.2 µM of each primer. Reactions were performed using T3000 thermal cycler (Analytik Jena, Jena, Germany) under the following conditions: an initial denaturation step at 95 °C for 3 min, followed by 30 cycles of denaturation (95 °C, 30 s), annealing (58 °C for ms172, ms207, ms203, ms213 and 60 °C for ms61, ms211, ms214, ms217, ms222, 30 s), extension (72 °C, 45 s) and final extension step (72 °C, 10 min). Electrophoresis was performed on 1,6% agarose gels at 70 V (2.4 V/cm) until the dye (bromophenol blue) reached 6 cm from the wells. Gels were stained in an ethidium bromide (EtBr) solution (0.5 µg/ml) for 10 min and destained in water for another 10 min. The gels were visualized under UV light using a FluorChem 8800 system with Alpha EaseFC v. 3.1.2 software (AlphaInnotech, San Leandro, CA, USA). For all analyzed VNTRs, a 100 bp Plus ladder size marker from MBI Fermentas was used. The size of each amplicon was measured with BioNumerics (version 4.6) software (Applied-Maths, Saint-Martens-Latem, Belgium). The sizes of PCR products were used for assessing the number of motif repeats. The repeat motif length and the 3’ and 5’ flanking sites were determined using the Vector NTI Advance Software v. 11.5.4 (ThermoFisher Scientific, Waltham, USA), according to Table 3. The number of mini- and microsatellite motif repeats was estimated by subtracting the invariable flanking region from the amplicon size and then dividing by the repeat unit length.

**Table 3:** The sizes of motif core and both 5’ and 3’ flanking sites.

VNTR	Repeated motif [bp]	5’ flank site [bp]	3’ flank site [bp]
ms061	6	25	35
ms172	54	185	195
ms211	101	138	105
ms213	103	61	118
ms214	115	133	67
ms217	109	165	204
ms222	101	128	73
ms207	6	70	45
ms209	6	70	43

### TRS-PCR genotyping and fingerprint analysis

Based on our preliminary results, the (CGG)_4_- and (GTC)_4_-based PCR fingerprinting were selected for the genotyping due to their highest differentiation rate among other analyzed trinucleotide repeat-based primers (data not published). 5’-N_6_(CGG)_4_ as well as 5’-N_6_(GTC)_4_ primers (N = A, T, C or G) were used in this study to amplify regions between loci complementary to trinucleotide repeat sequences. The reaction conditions for CGG-PCR were previously published elsewhere^19,20,22^. Similarly, the PCR mixtures for GTC-PCR (in a total volume of 50 µl) consisted of 20 ng of DNA, 1 × *Taq* polymerase reaction buffer (Invitrogen by Life Technologies), 1 U *Taq* polymerase (Invitrogen), 1.5 mM of MgCl_2_, 200 µM of each deoxynucleotide, 6% dimethyl sulfoxide (DMSO) and 0.1 µM of N_6_(GTC)_4_ primer. Reactions were performed using a T3000 thermal cycler under the following conditions: an initial denaturation step at 95 °C for 3 min, followed by 35 cycles of denaturation (95 °C, 1 min), annealing (57 °C, 1 min), extension (72 °C, 2 min) and final extension step (72 °C, 8 min). PCR amplicons were analyzed using the horizontal 1.6% agarose gel electrophoresis in a 1 × TAE buffer. A 100 bp Plus DNA size marker (Fermentas, ThermoFisher Scientific) was used to normalize the size of each PCR product.PCR amplicons were analyzed using the horizontal 1.6% agarose gel electrophoresis as described above. The cluster analyses of the TRS-PCR fingerprints were carried out with BioNumerics (version 4.6) software (Applied Maths, Sint-Martens-Latem, Belgium). The fingerprint similarity comparisons were calculated using a Pearson correlation (optimization 1%, position tolerance 1%), and grouping was performed according to the unweighted-pair group algorithm(UPGMA).TRS-PCR reproducibility was calculated by measuring the repeatability of TRS-PCR fingerprinting for three different *P. aeruginosa* isolates in quadruplicate (data not shown) using the SigmaPlot v14.0 (Systat Software Inc.). Strains were considered identical based on the reproducibility value of 93.3% (CGG-PCR) and 95.3% (GTC-PCR). Each individual TRS-PCR pattern was assigned a numerical value, as it was previously published^24^.

### Concordance of MLVA and TRS-PCR typing methods

The degree of congruence among MLVA and TRS-PCR typing schemes was determined using an online tool (http://www.comparingpartitions.info/; accessed on 09/06/2021). The probability that two strains classified as the same type by one method will also be classified as the same one using the other method is indicated by the Wallace coefficient (*W*). Intermethod concordance was calculated using the Wallace coefficient, as described by Wallace, D.L., 1983.

### Ethics committee approval

Consent of the Bioethics Committee KB74/2019 at the Institute of Tuberculosis and Lung Diseases regarding *Pseudomonas* strains isolated from cystic fibrosis patients includes.

- A statement confirming that all methods were carried out in accordance with relevant guidelines and regulations.
- A statement confirming that all experimental protocols have been approved by the designated institutional and/or licensing committee.
- Informed consent was obtained from all participants.

The consent was issued by: Bioethical Committee at the Institute of Tuberculosis and Lung Diseases, 26 Płocka Str., 01–138 Warsaw, Poland.

## Data availability

Raw data were generated at the Institute of Medical Biology of the Polish Academy of Sciences. Derived data supporting the findings of this study are available from the corresponding author on request.

## Abbreviations

CF: Cystic fibrosis
VNTR: Variable number tandem repeat
MLVA: Multiple loci VNTR analysis
CFTR: Cystic fibrosis transmembrane conductance regulator
TRS-PCR: Trinucleotide repeat sequences-polymerase chain reaction
SD: Standard deviation
IQR: Interquartile range
